# Binding Mechanisms of Intrinsically Disordered Proteins: Theory, Simulation, and Experiment

**DOI:** 10.3389/fmolb.2016.00052

**Published:** 2016-09-09

**Authors:** Luca Mollica, Luiza M. Bessa, Xavier Hanoulle, Malene Ringkjøbing Jensen, Martin Blackledge, Robert Schneider

**Affiliations:** ^1^CompuNet, Drug Discovery and Development, Istituto Italiano di TecnologiaGenova, Italy; ^2^NMR & Molecular Interactions, Université de Lille, CNRS, UMR 8576 - UGSF - Unité de Glycobiologie Structurale et FonctionnelleLille, France; ^3^Institut de Biologie Structurale, CEA, CNRS, Université Grenoble AlpesGrenoble, France

**Keywords:** intrinsically disordered proteins, protein-protein interactions, nuclear magnetic resonance, kinetics, molecular dynamics simulations

## Abstract

In recent years, protein science has been revolutionized by the discovery of intrinsically disordered proteins (IDPs). In contrast to the classical paradigm that a given protein sequence corresponds to a defined structure and an associated function, we now know that proteins can be functional in the absence of a stable three-dimensional structure. In many cases, disordered proteins or protein regions become structured, at least locally, upon interacting with their physiological partners. Many, sometimes conflicting, hypotheses have been put forward regarding the interaction mechanisms of IDPs and the potential advantages of disorder for protein-protein interactions. Whether disorder may increase, as proposed, e.g., in the “fly-casting” hypothesis, or decrease binding rates, increase or decrease binding specificity, or what role pre-formed structure might play in interactions involving IDPs (conformational selection vs. induced fit), are subjects of intense debate. Experimentally, these questions remain difficult to address. Here, we review experimental studies of binding mechanisms of IDPs using NMR spectroscopy and transient kinetic techniques, as well as the underlying theoretical concepts and numerical methods that can be applied to describe these interactions at the atomic level. The available literature suggests that the kinetic and thermodynamic parameters characterizing interactions involving IDPs can vary widely and that there may be no single common mechanism that can explain the different binding modes observed experimentally. Rather, disordered proteins appear to make combined use of features such as pre-formed structure and flexibility, depending on the individual system and the functional context.

## Introduction

The discovery of intrinsically disordered proteins (IDPs) has considerably enhanced our view of protein structure and function. Over the last two decades, it has become accepted that proteins can be functional in the absence of a stable three-dimensional structure (Wright and Dyson, 1999; Dunker et al., 2001, 2002; Tompa, 2002; Dyson and Wright, 2005), and more recently, it has been shown that intrinsic disorder is compatible with the environment inside the cell (Bodart et al., 2008; Theillet et al., 2016). Bioinformatic predictions estimate that on the order of 30% of eukaryotic proteins contain disordered regions of sizable length (>=50 residues; Dunker et al., 2000), and the DisProt database of protein disorder (http://www.disprot.org/) now contains entries for 1539 disordered protein regions and 694 entirely disordered proteins (Sickmeier et al., 2007). Their abundance, as well as their implication in disease (Uversky et al., 2008), has sparked considerable interest in IDPs, and a large number of studies have been devoted to the development of experimental and computational approaches in order to understand their conformational behavior and molecular function (Jensen et al., 2014).

IDPs are implicated in a wide range of biological functions, among them notably signal transduction, scaffolding, transcription, cell cycle regulation, or chaperoning (Dunker et al., 2002; Dyson and Wright, 2005). A common theme to these functions are interactions with other proteins or alternatively DNA or small molecules. Given the apparent frequency with which disorder occurs in the aforementioned functional contexts, questions arise as to how IDPs interact with their partners in the absence of well-structured binding sites, which mechanisms they employ to assure specific binding, and in general the advantages intrinsic disorder may have for protein-protein interactions.

**Figure 1:**
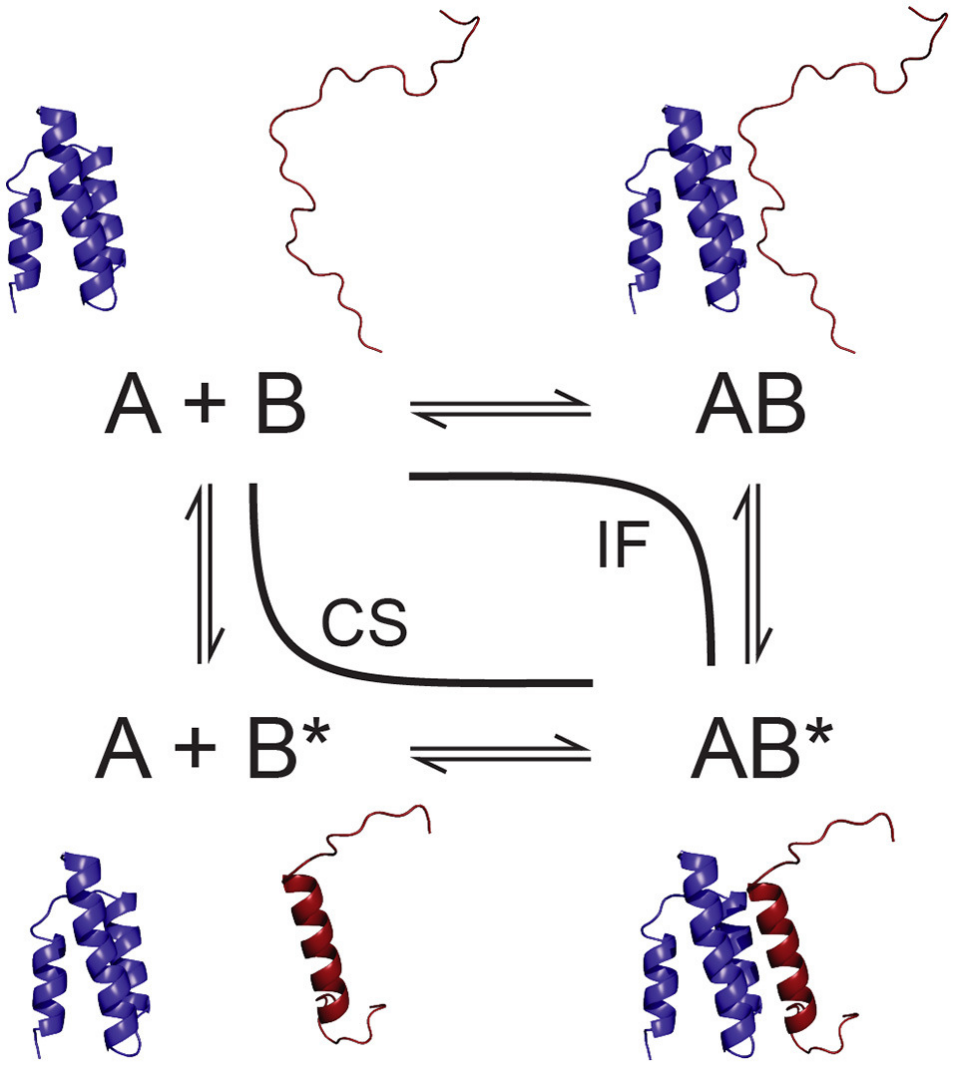
**Schematic of conformational selection (CS) and induced fit (IF) as parallel pathways of a bimolecular folding and binding interaction**. One of the partners, B, is an IDP that exists in different conformations, unfolded (B) and folded into its final bound state (B^*^).

In many cases, IDPs do not exhibit fully random statistical coil behavior, but can adopt transiently populated secondary structures and long-range tertiary interactions (Fuxreiter et al., 2004; Salmon et al., 2010). Such structural preorganization has often been found relevant in intermolecular interactions undergone by IDPs. While so-called “fuzzy complexes” of IDPs have been described that retain a high degree of disorder even in the bound state (Tompa and Fuxreiter, 2008), binding of IDPs to physiological partners is often accompanied by a gain in structuration of the binding region, a phenomenon known as “folding upon binding” or “coupled folding and binding” (Dyson and Wright, 2002). How exactly this is accomplished mechanistically has been the subject of intense debate. The discussion has mostly focused on two mechanisms that can be considered as limiting cases, conformational selection (i.e., folding before binding; Karush, 1950; Ma et al., 1999) and induced fit (i.e., folding after binding; Koshland, 1958; Figure 1), while more complex mechanisms such as different combinations of these two have also been envisioned (Csermely et al., 2010). In parallel, other, partially related questions have been discussed, such as whether increasing disorder speeds up binding, as proposed in the often-cited “fly-casting” hypothesis (Shoemaker et al., 2000), or conversely, whether increased pre-structuration of IDP binding sites allows for faster binding (Iešmantavičius et al., 2014). Another matter of debate concerns binding affinities and binding specificity achievable in the context of intrinsic disorder, with IDPs often said to accomplish highly specific binding without concomitant high affinity, which may be of advantage in the context of signaling where a once-formed complex needs to dissociate again to switch off the corresponding signal (Tompa, 2002; Zhou, 2012).

Answering these questions is not a trivial task, and especially obtaining detailed mechanistic information on binding interactions, which usually requires the extraction of the rate constants involved, is notoriously difficult. Correspondingly, the number of experimental studies explicitly addressing IDP binding mechanisms is still relatively limited. Nevertheless, recent years have seen a steady increase of research into the details of IDP binding interactions. Two experimental techniques have in particular been used, namely nuclear magnetic resonance (NMR) spectroscopy and non-equilibrium transient kinetic techniques, such as stopped flow or temperature jump experiments (Gianni et al., 2016). Surface plasmon resonance (SPR) measurements also allow for detailed insight into binding kinetics at high temporal resolution, however at the price of immobilization of one of the binding partners, which may affect the interaction in the case of a highly flexible IDP (Schuck and Zhao, 2010). In addition, single-molecule experiments such as fluorescence resonance energy transfer (Gambin et al., 2011) or nanopore translocation (Japrung et al., 2013) have recently gained importance in studying binding reactions. In parallel to these experimental techniques, modeling and computer simulations have increasingly contributed to our understanding of protein interactions at the atomic level, notably also due to the development of various enhanced sampling techniques (Zhou and Bates, 2013; De Vivo et al., 2016). Here, we have chosen to review recent progress in the field based on NMR spectroscopy and transient kinetics experiments, as well as molecular simulations.

## Methodology

### Transient kinetics

**Figure 2:**
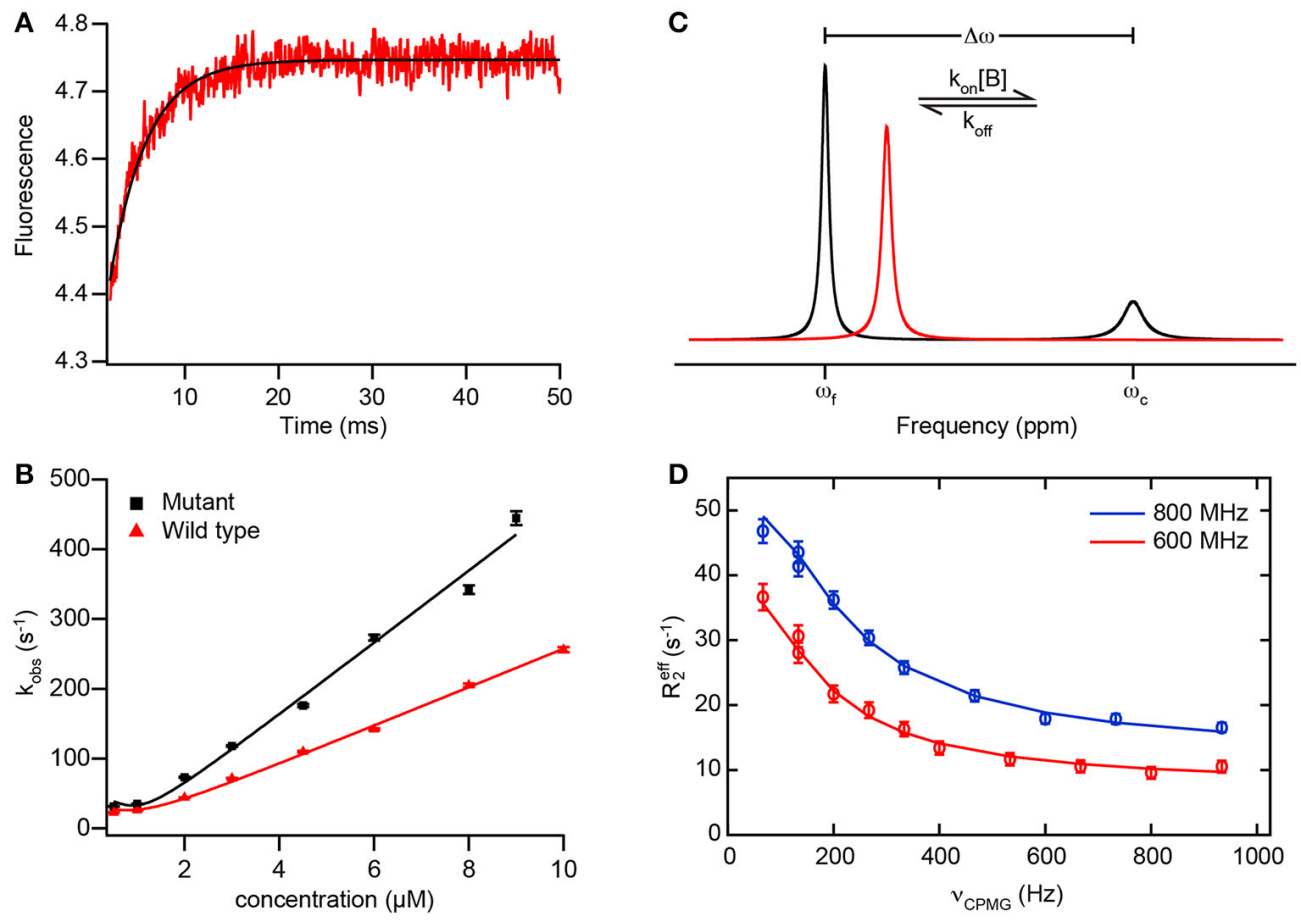
**Example data from transient kinetics and NMR to analyze binding mechanisms. (A)** Fluorescence trace of a binding reaction measured in a stopped-flow experiment. The fluorescence of an introduced tryptophan residue (W2108) of NCBD(Y2108W) is monitored upon binding of disordered ACTR (see text for details). Red, experimental data; black, fit of an exponential function to obtain *k*_obs_. **(B)** Dependence of the rate constant *k*_obs_ observed in experiments as depicted in **(A)** with varying concentrations of wild-type ACTR (red) and a mutant ACTR variant with increased helix propensity (black). Solid lines are fits using the general equation for reversible association of two molecules (valid also for non-pseudo-first order conditions; Malatesta, 2005). **(A,B)** adapted from Iešmantavičius et al. (2014) with permission of John Wiley & Sons, Inc. **(C)** Schematic 1D NMR spectra of a spin undergoing exchange between two states with different chemical shifts, such as in a reversible binding interaction as in Equation (1). The spin is assumed to be in molecule A, such that the effective association rate constant is given by *k*_on_[B]. The free state (chemical shift ω_f_) is assumed to be dominant and the complex (chemical shift ω_c_) a minor state. Black, spectrum for intermediate to slow exchange (*k*_ex_ < Δω) with two resolved resonance lines for free and bound state; red, spectrum for intermediate to fast exchange (*k*_ex_ > Δω) with one averaged resonance signal. In both cases, the effective transverse relaxation rate (and thus the linewidth) of the signals contains a contribution from the exchange, leading to additional line broadening. Note that the minor signal in the black spectrum is preferentially broadened due to its larger exchange contribution *k*_off_, which can lead to broadening beyond detection. **(D)** Example data from a CPMG relaxation dispersion experiment measured at two static magnetic fields (red, 600 MHz, blue, 800 MHz ^1^H Larmor frequency) on the carbonyl ^13^C of residue 475 in the PX binding site of Sendai virus N_TAIL_ in the presence of 8% (molar) of PX (see text for details; Schneider et al., 2015). Data points show the effective transverse relaxation rate *R*_2, eff_ of the visible major (free) state signal for different CPMG pulse frequencies ν_CPMG_. Solid lines are fits to the data using a model of exchange between three states, corresponding to free N_TAIL_, encounter and final complex, allowing for extraction of the rate constants, populations, and chemical shift differences along the interaction trajectory.

Kinetic information on a binding interaction can be obtained from non-equilibrium techniques that either rapidly mix the reactants (e.g., stopped or continuous flow) or perturb a preexisting equilibrium between them, for example by application of a rapid temperature or pressure jump, and then follow the (re-)establishment of equilibrium via a signal whose variation is related to the binding reaction. Usually, optical signals are employed, such as circular dichroism (CD), absorbance, or the fluorescence of a native or introduced aromatic residue, which vary in the course of a folding and/or binding reaction (Bernasconi, 1976; Figure 2A). These techniques, originally developed in the context of the study of enzymatic reactions (Eigen and Hammes, 1963), are sensitive to processes occurring on the timescales of microseconds (temperature or pressure jump, continuous flow) to milliseconds and longer (stopped flow) (Gianni et al., 2016). Depending on the relaxation time course of the signal monitored, one or more kinetic time constants (λ or *k*_obs_) are obtained from fitting the signal decay. For a simple two-state bimolecular binding reaction with 1:1 stoichiometry

(1) $$\text{A}+\text{B}\overset{k_{\text{on}}}{\underset{k_{\text{off}}}{⇌}}\text{AB}$$

assuming pseudo-first-order conditions for A (i.e., B is present in excess), *k*_obs_ is given by the sum of the forward and reverse reaction rate constants, *k*_on_[B] + *k*_off_. A multiexponential decay, on the other hand, is direct evidence for a more complex binding mechanism (Kiefhaber et al., 2012; Vogt and Di Cera, 2012). The reaction is then followed over a range of concentrations of the binding partner in excess. Fitting appropriate models to the variation of the observed relaxation rate constant(s) with binding partner concentration allows for the extraction of the underlying reaction rate constants (Figure 2B). Notably, even if only one rate constant *k*_obs_ is experimentally observable, a nonlinear variation of *k*_obs_ with ligand concentration is evidence for a multistep binding mechanism, and the exact dependence of *k*_obs_ on concentration gives information on its nature (Tummino and Copeland, 2008; Vogt and Di Cera, 2012). A shortcoming of these methods is that they do not offer site-specific resolution, and the exact nature of the event leading to a change in the observed signal is usually not known.

### NMR spectroscopy

NMR spectroscopy is a sensitive method to investigate binding reactions in proteins and can yield residue-specific information on binding sites, affinities and mechanisms (Zuiderweg, 2002). Typically, binding is monitored by observing the NMR resonances of an isotope- (^15^N- and/or ^13^C-) labeled protein in the presence of unlabeled, i.e., NMR-inactive ligand. Ligand binding induces changes in the magnetic environment of spins in the residues involved in the interaction, reflected in changes in the chemical shifts of NMR resonances and/or their line shapes. Importantly, the underlying dynamics of the exchange of a spin between free and bound states affect NMR observables and can be measured using suitable experiments (Mittermaier and Kay, 2009). Thus, although NMR is an equilibrium technique, it can nevertheless provide access to kinetic details of a binding reaction and thereby its mechanism.

How NMR chemical shifts are affected by titrating with increasing amounts of ligand depends on the underlying exchange rate *k*_ex_ between free and bound state, which, for the simple two-state binding scheme of Equation (1), is equal to the *k*_obs_ observed in kinetic experiments (*k*_on_[B] + *k*_off_ for the exchange rate relevant if molecule A is observed). In the so-called fast exchange regime, i.e., if *k*_ex_ is much larger than the chemical shift difference between free- and bound-state signals (*k*_ex_ ≫ Δω), a single resonance peak is observed for a given spin which moves from the free-state to the bound-state position in the course of the titration. Conversely, for slow exchange (*k*_ex_ ≪ Δω), the free-state resonances disappear upon titration, while the bound-state signals progressively appear in the spectrum (Figure 2C). For intermediate exchange (*k*_ex_ ≈ Δω), a combination of the two phenomena may be observed, but usually strong broadening or even disappearance of resonance signals occurs (Mittermaier and Kay, 2009). Notably, such titration experiments can already point to the presence of more complex binding mechanisms via, for example, peaks following a curved path (Arai et al., 2012), or combinations of fast- and slow-exchange behavior in individual resonances (Sugase et al., 2007a). Peaks that remain exchange-broadened even in the presence of excess ligand have also been observed in IDP interactions with their partners (Jensen et al., 2011; Schneider et al., 2015), suggesting that the interaction kinetics are characterized by more than two states, for example if the bound state exhibits additional dynamics on the μs–ms timescale.

On the timescales typical for protein-protein binding (μs–ms range), NMR experiments useful for characterizing the exchange underlying such spectral changes are rotating-frame (R_1ρ_; Palmer and Massi, 2006) and Carr-Purcell-Meiboom-Gill (CPMG) relaxation dispersion (Palmer et al., 2001) as well as chemical exchange saturation transfer (CEST) experiments (Vallurupalli et al., 2012). In the context of analyzing IDP binding interactions, especially CPMG relaxation dispersion has been used. This experiment is normally conducted under conditions where either the free or the bound state of the protein under study is dominant and the respective other state(s) spectrally invisible due to its/their low population and preferential exchange broadening. If the exchange between free and bound states occurs on a timescale between about 100 and 2500 s^−1^, its contribution to the effective transverse relaxation rate *R*_2, eff_ (and thus the linewidth) of the visible NMR signals can be quenched by the application of a train of 180° pulses of increasing frequency ν_CPMG_ (Mittermaier and Kay, 2009). Fitting the resultant dependence of *R*_2, eff_ on ν_CPMG_ to a model of the underlying exchange yields its exchange rate(s) *k*_ex_, the populations of the states involved, and the chemical shift differences between them (Figure 2D). Individual rate constants *k*_on_ and *k*_off_ may be extracted from *k*_ex_ by measuring relaxation dispersions at different concentrations or temperatures, or by knowledge of the dissociation constant, *K*_d_, of the interaction. In practice, the complexity of systems that can usefully be addressed using this strategy is limited to three exchanging states. NMR signals are broadened by exchange on a similar timescale as the one CPMG experiments are sensitive to (μs–ms); consequently, line broadening may hamper data analysis.

### Molecular simulations

Simulations can, in principle, visualize biomolecular function and interactions in atomic detail and thus allow to elucidate the underlying mechanisms that are often difficult to access from experimental data alone. Recent years have seen a steady increase in both computing power and availability of resources for simulation techniques, from in-house clusters and graphical processing unit- (GPU-) based algorithms to large-scale computing facilities. However, both the models and parameters used as well as the simulation results need to be calibrated against and verified by experimental data to ensure that meaningful results are obtained. IDP systems pose some particular challenges for simulations. Due to their conformational heterogeneity, extensive sampling is required to ensure that the conformational space is adequately covered by the simulation. In addition, compared to their large number of degrees of freedom, experimental data on IDPs is typically sparse, i.e., their characterization is an underdetermined problem and care must be taken, e.g., by cross-validation approaches, to avoid overfitting. Finally, the accuracy of modern force fields for IDP simulations is not well characterized, and standard combinations of force fields and water models primarily developed for folded proteins may not be appropriate for simulations of IDP systems.

Standard molecular dynamics (MD) simulations of IDPs, using all-atom or united-atom representations (the latter with most nonpolar hydrogen atoms “collapsed” into the heavy atom to which they are bound), typically require long simulation times on the microsecond timescale to yield reasonable agreement with experimental data (Lindorff-Larsen et al., 2012). Most commonly, variants of the quantum mechanics-based AMBER or the empirically parametrized CHARMM and GROMOS force fields are used. Some variants have been optimized against NMR parameters, such as AMBER ff99SB-ILDN (Lindorff-Larsen et al., 2010) or CHARMM22^*^ (Best and Hummer, 2009), or improved directly using NMR observables, like AMBER ff99SBNMR1-ILDN, which also contains improved representations of torsion angle potentials (Li and Brüschweiler, 2010). Insufficient solvation of proteins has been observed using current force fields, which is particularly problematic for IDPs (Mercadante et al., 2015). To address this problem, short-range protein-water interactions can be modified in current water models such as TIP3P or SPC known to accurately reproduce the structure of liquid water (Horn et al., 2004; Wang L.-P et al., 2014; Henriques et al., 2015); alternatively, a new water model, TIP4P-D, has been proposed to correct for underestimation of London dispersion interactions (Piana et al., 2015). Extensive simulations on disordered peptides and validation against experimental NMR and small-angle X-ray scattering (SAXS) data have indicated that CHARM22^*^ in conjunction with the TIP4P-D water model reproduces experimental data well, with the notable exception of NMR residual dipolar couplings (RDCs; Rauscher et al., 2015). This shortcoming has been observed for all available force fields and is likely due to the sensitivity of RDCs to both local and long-range structuration. Correspondingly, a bias in current force fields toward overly compact structures—as measured by, e.g., radius of gyration—has been observed (Henriques et al., 2015), which may partly explain why these force fields perform well for compact, folded proteins, but less so for simulations of IDPs where correct reproduction of their flexible and extended nature is essential. The TIP4P-D water model has also been observed to overly destabilize transient secondary structure (Salvi et al., 2016). Efforts have been made to correct for these deficiencies, with the specific aim to better reproduce IDP conformational sampling, for example by corrections to the dihedral potentials of disorder-promoting residues in AMBER ff99SB-ILDN, with some improvement in reproduction of NMR chemical shifts (Wang W. et al., 2014; Ye et al., 2015). Nevertheless, it appears that current force fields and water models are still not calibrated accurately enough to fully capture conformational sampling in IDPs, especially in the presence of transient structure, warranting caution in the interpretation of IDP simulations in terms of underlying mechanisms.

Coupled folding and binding of IDPs to their partners is typically still out of reach of classical MD simulations due to the large number of degrees of freedom as well as the extensive conformational transitions involved. To overcome the sampling limit of classical MD, different approaches can be used. Coarse-grained (CG) simulations reduce the number of degrees of freedom by simplified representations of the molecular system, parametrizing individual amino acids using only one or a few hard spheres (Takada et al., 2015). Compared to all-atom simulations, this yields better agreement of the radius of gyration of IDPs with experiment (Smith et al., 2014). CG simulations are often combined with empirical “Gō-type” potentials to simulate protein-protein binding (Ueda et al., 1978; Karanicolas and Brooks, 2002). Here, terms for non-bonded interactions can be included which favor contacts between those residue pairs that are in contact in a known native structure of the complex investigated (De Sancho and Best, 2012). For protein binding or unfolding, Monte Carlo (MC) simulations have also been used. Here, the properties of the system are computed by repeated random sampling whose outcome is periodically accepted or rejected on the basis of a deterministic principle, i.e., among the conformations of a molecule corresponding to two consecutive steps, the one with minimal energy is chosen (Metropolis criterion; Irbäck and Mohanty, 2006; Staneva et al., 2012).

On the other hand, various enhanced sampling techniques for MD simulations have been proposed. One of the most widely used methods is parallel tempering or replica exchange molecular dynamics (REMD; Okamoto, 2004; Tai, 2004; Ostermeir and Zacharias, 2013). Here, several copies (replicas) of the system are simulated independently and simultaneously at different simulation temperatures. At preset intervals, pairs of replicas are exchanged with a specified transition probability. This allows the system to escape from being trapped in locally stable states at low simulation temperature, while preserving the canonical distribution of sampled states within each replica. Instead of using the simulation temperature as replica co-ordinate (T-REMD), it is also possible to vary the force field or system Hamiltonian across replicas. While improving sampling, REMD is computationally expensive and can become prohibitive for large proteins. An alternative approach known as metadynamics is based on the dynamics performed by a few collective variables of the atomic coordinates (Laio and Parrinello, 2002). In a metadynamics simulation, the system is driven not only by its potential energy, but also by a biasing history-dependent potential constructed as a sum of user-defined Gaussians centered along the trajectory of the collective variables which “fill” the free-energy surface to drive the system away from states that were already visited. Two variants of this technique have mostly been used: well-tempered metadynamics (W-META), a variant that adds an adaptive bias along the trajectory of the collective variables by varying the Gaussian height (Barducci et al., 2008); and bias exchange metadynamics (BE-META), which combines standard metadynamics with the replica exchange approach, allowing for extensive sampling of the free energy landscape (Domene et al., 2015). Recent studies have demonstrated that both BE-META and T-REMD are capable of reproducing secondary structure and lowly populated conformations of disordered peptides (Do et al., 2014; Zerze et al., 2015).

## Possible advantages of intrinsic disorder

The possible consequences and advantages of intrinsic disorder for protein function and interactions have been extensively discussed (Dunker et al., 2002; Tompa, 2002; Dyson and Wright, 2005; Wright and Dyson, 2015). It appears that the specific qualities of disorder are well suited for the functions in interaction and regulation frequently fulfilled by IDPs. For example, their extended, flexible nature may be advantageous for the assembly of complexes due to facile access to the binding sites on an IDP and lack of steric hindrance (Tompa, 2002). Similarly, disordered regions can easily be accessed by modifying enzymes such as kinases, which may be why posttranslational modification sites are also often found in disordered regions (Iakoucheva et al., 2004). At the same time, disordered proteins can form extended binding interfaces that are large compared to their own size, offering the possibility of specific, high-affinity partner recognition (Mészáros et al., 2007; Dogan et al., 2014). On the other hand, their plasticity allows them to bind to different partners, using different or even identical binding sites, a phenomenon known as promiscuity (Tompa et al., 2005; Oldfield et al., 2008). Furthermore, turnover of IDPs in the cell is usually more rapid due to their susceptibility to proteases, which may be of advantage in signaling (Wright and Dyson, 1999; Dyson and Wright, 2005).

Two possible advantages of intrinsic disorder for protein-protein interactions have been put forward and discussed particularly frequently: an increased association rate (*k*_on_) due to a “fly-casting” effect (Shoemaker et al., 2000), and an ability to achieve high binding specificity without concomitant high affinity (Zhou, 2012). We will look at these two propositions in more detail in the following sections.

### High association rates: fly casting vs. pre-formed structure

The original proposition of the “fly-casting” effect stated that an unfolded protein should be able to form initial interactions with its binding partner already at a greater distance than a folded protein, leading to an increased capture radius and a “reeling in” of the partner to its binding site on the IDP (or vice versa; Shoemaker et al., 2000). This was predicted to lead to an, actually relatively modest, increase in the association rate by a factor of 1.6; however, larger rate enhancements have been suggested for other, more realistic models than were used in the original derivation (Shoemaker et al., 2000; Zhou et al., 2012). Experimentally, very fast association rates around the estimated diffusion limit for folded proteins (up to about 10^9^ –10^10^ M^−1^s^−1^) are found surprisingly frequently for IDPs (Sugase et al., 2007b; Arai et al., 2012; Shammas et al., 2013; Dogan et al., 2015; Milles et al., 2015). Association rates exceeding 10^7^ M^−1^s^−1^ are invariably associated with electrostatic attraction (Zhou and Bates, 2013). Indeed, IDPs in general tend to exhibit an elevated net charge (Uversky, 2002), and the vicinities of IDP binding sites have been described to be enriched in complementary charges (Ganguly et al., 2012). However, even if corrections for the effects of electrostatic attraction are applied, *k*_on_ values in the diffusion-limited range have been found for IDPs (10^5^ –10^6^ M^−1^s^−1^) (Shammas et al., 2013; Dogan et al., 2015; Milles et al., 2015). This suggests that disorder *per se* may indeed speed up the association process, via fly casting or otherwise. However, the number of kinetic studies of IDP association reactions is still relatively limited, and overall, a wide range of association rate constants has been measured for IDPs, similar to what has been observed for folded proteins (Dogan et al., 2014). Thus, so far it does not seem warranted to claim that IDPs can generally achieve faster association than folded proteins.

Fly casting is difficult to prove or disprove experimentally, largely owing to the difficulty of changing the level of disorder in one of the partners of a binding interaction without affecting other factors as well. Note that the original publication considered a comparison between a completely and a partly folded protein to derive the prediction of a 1.6-fold increase in *k*_on_. However, most experimental assessments which can be related to the fly-casting hypothesis have rather involved short binding sites of IDPs exhibiting partial order, such as a transient helix, and modified the propensity to form such structure via mutations or cosolvents. In most of these experiments, stabilizing (secondary) structure was however found to increase association rates, albeit to different extent (Rogers et al., 2013, 2014b; Iešmantavičius et al., 2014; Arai et al., 2015), contradicting the fly-casting hypothesis. The association of the p27 cyclin-dependent kinase (CDK) inhibitor with the cyclin A–Cdk2 complex appears to be an exception; here, stabilizing the linker helix slowed down the time course of inhibition (Bienkiewicz et al., 2002). However, the mutations employed also changed the charge of p27. Moreover, the linker helix makes only few contacts with the cyclin A–Cdk2 complex and likely functions mostly as a spacer between the cyclin A- and Cdk2-binding domains of p27 (Lacy et al., 2004). Investigations of the kinetics of different proteins, with different degrees of disorder, binding to a single target have suggested no influence of pre-formed structure on association rates, or rather kinetic advantages for the more disordered proteins (Shammas et al., 2014; Dogan et al., 2015); however, comparing different proteins with regard to an individual quality is inherently difficult. Thus, while the experimental evidence is not entirely conclusive in this regard, it appears nevertheless that stabilization of the bound-state structure in an IDP prior to interaction leads to faster association rates.

CG simulations with a Gō-like potential of the interaction of the phosphorylated kinase inducible domain (pKID) of the cAMP response element binding protein (CREB) with the KIX domain of the CREB-binding protein (CBP) have also suggested that the kinetic advantage of an increased capture radius would be offset by the slower translational diffusion of a disordered protein (Huang and Liu, 2009). This likely only applies to large-scale changes in disorder, and thus diffusion coefficient, envisioned in the original fly-casting hypothesis; stabilization of a transient helix in the binding site of an IDP was not observed to have an effect on its diffusion (Iešmantavičius et al., 2014). However, based on these simulations, another mechanism was proposed by which disorder could speed up protein-protein association, namely a reduction of the free-energy barrier between initial and final complex due to facilitated folding after initial binding (i.e., induced fit, see also below), or equivalently, an increase in the number of collisions leading to productive binding (Huang and Liu, 2009). The expected increase in *k*_on_ from this effect is again rather modest, a factor of 2.5 (Huang and Liu, 2009). Similarly, in Gō model simulations on the interaction of the C-TAD domain of hypoxia-inducible factor-1α (HIF1α) to the TAZ1 domain of CBP, the observed association rate approached the fast experimental one only if the model allowed for non-native contacts to occur, leading to a broad distribution of encounter complexes productive for binding (De Sancho and Best, 2012). Electrostatic interactions appear to play a crucial role in this process by reducing the redissociation rate after initial encounter, as well as by increasing the probability of native-like topologies in the collision complexes (Ganguly et al., 2013). A facilitated transition between initial and final complex bears some resemblance to a fly-casting mechanism, albeit without an effect of a larger capture radius. Notably, rather than increasing association rates beyond those achievable by ordered proteins, this effect of disorder seems to avoid the orientational restraints and steric hindrance that would result if IDPs had to rigidly dock their binding partners, at least in those cases where the binding interface is more complex and extended (Zhou et al., 2012). In the aforementioned case of p27 binding to the cyclin A–Cdk2 complex, it was noted that, due to steric clashes, p27 could not bind if it were rigid in its bound conformation; this would explain the requirement for a flexible linker helix found experimentally (Bienkiewicz et al., 2002; Zhou et al., 2012). One could thus argue that the flexibility of IDPs counterbalances the negative effects on association rates that their often extended binding interfaces would otherwise have, globally leading to a similar range of association rates as found in folded proteins (Dogan et al., 2014).

Regardless of the effects of pre-formed structure on kinetics, stabilization of bound-state secondary structure has always been observed to increase affinity and lead to corresponding changes in downstream functional responses of IDPs (Parker et al., 1998, 1999; Petros et al., 2000; Rogers et al., 2014b). For example, stabilizing the transient helix with which the transactivation domain (TAD) of the tumor suppressor p53 binds to the repressor oncoprotein MDM2 leads to stronger binding and concomitant effects on target gene expression and cell cycle regulation (Zondlo et al., 2006; Li et al., 2010; Borcherds et al., 2014). In the disordered domain D2 of hepatitis C virus (HCV) protein NS5A, a short structural motif dubbed the PW turn was identified whose disruption abolished HCV replication and affected interaction with the host protein cyclophilin A (Dujardin et al., 2015). These and other examples underline the importance of pre-formed structure in IDP binding and indicate that it typically has a functional role.

### Affinity and specificity

It has been suggested that the loss of conformational entropy of IDPs associated with folding upon binding results in an overall unfavorable entropic contribution to binding, thereby uncoupling binding strength from specificity, i.e., allowing for highly specific binding without high affinity (Tompa, 2002; Fuxreiter et al., 2004; Dyson and Wright, 2005; Zhou, 2012). This has been cited as an example of enthalpy-entropy compensation (Fuxreiter et al., 2004) and as an advantage in the context of signaling, where interactions have to be reversible to assure that a signal can be switched off again. However, it is not entirely clear how disorder can uncouple specificity and affinity, since the two are intimately linked by definition. Thermodynamic specificity, in essence, consists in differences in (relative) affinity to multiple possible targets and therefore also depends on the availability and concentration of binding partners (Zondlo et al., 2006). Thus, an interaction of weak or moderate affinity may well be highly specific, however only as long as any possible competing interaction exhibits even weaker affinity (or the concentrations of other binding partners are sufficiently low). Also, in principle, if a binding interaction is accompanied by a large unfavorable entropy change, and if enthalpy-entropy compensation is at play, the result should not be an overall low affinity of the complex, but rather a concomitant large favorable enthalpic contribution, as has been noted in the context of folding upon binding in protein-DNA interactions (Spolar and Record, 1994). Finally, IDPs also engage in promiscuous interactions with different binding partners, even via identical binding sites as in the case of the C-terminal regulatory region of p53 (Oldfield et al., 2008), possibly leading to different functional responses (moonlighting; Tompa et al., 2005). It is not clear how intrinsic disorder would be able to both assure high binding specificity and still permit promiscuous interactions.

In fact, the reduction in entropy associated with folding upon binding of an IDP does not generally entail an overall unfavorable entropic contribution to binding. First, pre-formed structure corresponding to the bound conformation, as well as disordered segments not directly involved in the binding, reduce the entropy penalty of folding upon binding (Fuxreiter et al., 2004), as is the case for so-called “fuzzy complexes” that retain dynamics even within the binding regions (Mittag et al., 2008; Tompa and Fuxreiter, 2008). But even for IDP complexes reaching a stable bound conformation, the gain in solvent entropy from the release of solvation water molecules can be considerable, especially given that IDPs tend to form comparatively large, extended binding interfaces with pronounced hydrophobic character (Dyson and Wright, 2005; Mészáros et al., 2007; Ganguly et al., 2012; Dogan et al., 2014). The overall entropy of folding and binding of an IDP to its partner may thus well be favorable, as, for example, in the case of the interaction of the disordered transactivation domain of the transcriptional activator c-Myb with the KIX domain of CBP (Parker et al., 1999). Notably, the formation of a large hydrophobic interface typically also leads to a favorable binding enthalpy, which can compensate for unfavorable entropic contributions, as seen for example in the interaction of the disordered activation domain of the activator for thyroid hormone and retinoid receptors (ACTR) with the nuclear coactivator binding domain (NCBD) of CBP (Demarest et al., 2002).

Overall, as is the case for association rates, IDP interactions exhibit a wide range of affinities, including very high ones, as do folded proteins (Dogan et al., 2014). Notably, nanomolar dissociation constants have been found even for IDP complexes involved in signaling, such as ACTR–NCBD (Demarest et al., 2002), p27–cyclin A–Cdk2 (Lacy et al., 2004), or the complex between disordered PUMA and folded Mcl-1 from the mammalian Bcl-2 family of apoptosis regulators (Rogers et al., 2014b). How the signal associated with the formation of such complexes is switched off again, given their high affinity, is not clear. However, other, competing interactions of comparable affinity may be involved. As mentioned, specificity essentially corresponds to relative affinity and thus depends on the context in which a particular complex exists. In any case, intrinsic disorder does not seem to generally lead to low-affinity and/or high-specificity complexes.

## Conformational selection, induced fit and beyond

### Distinguishing conformational selection and induced fit

The discussion about the mechanisms used by IDPs to bind to their partners has often been framed in the context of a supposed dichotomy of conformational selection vs. induced fit (Figure 1), while more recent results tend to suggest that IDP binding mechanisms may be more complex, exhibiting various mixed, intermediate mechanisms, or a coexistence of these two limiting cases (Espinoza-Fonseca, 2009; Csermely et al., 2010). Conformational selection, in a strict sense, implies that the bound-state conformation of an IDP preexists in its free state and that exclusively this conformation is binding competent. Induced fit in the context of IDPs, on the other hand, entails that binding occurs in a more or less unfolded state and that conformational rearrangements to the final bound state take place in a unimolecular reaction within the complex. Evidence for both mechanisms has been found in binding of various IDPs to their partners, sometimes even for a single IDP (Gianni et al., 2012; Arai et al., 2015), but conclusive proof of one or the other is usually difficult and may not reflect the true complexity of the underlying binding mechanism.

As mentioned, a necessary condition for a conformational selection mechanism is that molecules assuming the bound conformation preexist in the free state ensemble of the IDP. NMR and MD studies have often found evidence for such pre-formed conformers closely resembling the bound state, for IDPs as well as for folded proteins, suggesting that conformational selection plays a role in their binding (Lange et al., 2008; Boehr et al., 2009; Kjaergaard et al., 2010; Jensen et al., 2011; Krieger et al., 2014). However, pre-formed bound-state structure *per se* is not evidence for a conformational selection mechanism, since its existence does not prove its (exclusive) implication in binding (Dogan and Jemth, 2014). Such evidence normally requires kinetic measurements of the reaction rate constants over a range of concentrations of at least one of the binding partners. However, in many cases, multistep reactions such as folding before or after binding do not become directly evident as, for example, multiexponential relaxation in non-equilibrium experiments or evident three-state behavior in NMR relaxation dispersion data.

For transient kinetics experiments, even if only a single *k*_obs_ is observable experimentally, a multistep reaction can nevertheless become apparent if *k*_obs_ varies nonlinearly with the concentration of the binding partner. In that case, a *k*_obs_ increasing or decreasing hyperbolically with concentration has often been taken as evidence for an induced fit or conformational selection mechanism, respectively (Tummino and Copeland, 2008). However, this is only valid under the so-called rapid equilibrium assumption, where binding is assumed to be fast relative to the folding step (Vogt and Di Cera, 2012). This assumption is not necessarily justified, especially in the context of IDPs forming short elements of secondary structure, which can occur on a sub-μs timescale (Eaton et al., 1997). Consequently, it has been shown that, while a *k*_obs_ decreasing with binding partner concentration can only be explained by conformational selection, a hyperbolic increase in *k*_obs_, as is often observed experimentally, can actually be accounted for by both induced fit and conformational selection mechanisms, leading to the suggestion that binding by conformational selection may be much more widespread than previously thought (Vogt and Di Cera, 2012, 2013). Recently, it has been pointed out that a distinction between the two binding mechanisms may be made in kinetic experiments by varying the concentrations of both reaction partners in separate experiments (Gianni et al., 2014). Induced fit yields a hyperbolically increasing *k*_obs_ in both cases. Conformational selection, on the other hand, results in a linear *k*_obs_ variation if the concentration of the species undergoing conformational change is varied, while hyperbolic behavior (increasing or decreasing *k*_obs_) is retained for variation of the concentration of the other partner. In practice, it may however be difficult to obtain data for a wide enough range of protein concentrations to draw clear mechanistic conclusions. Alternatively, binding kinetics may be studied at different temperatures or with different ligands; however, the latter method assumes that only the microscopic rate constants, not the basic mechanism, would change for different ligands (Vogt and Di Cera, 2012).

### Conundrums in experimental data

Kinetic data that can satisfactorily be explained by conformational selection or induced fit alone have been described in the literature, often for enzymes binding to their substrates (Kirschner et al., 1966; Wong et al., 1991; Kim et al., 2007; Johnson, 2008). Frequently, however, NMR or non-equilibrium kinetics measurements of IDP binding interactions give no direct indications of multistep mechanisms at all, with NMR relaxation dispersion data being well fit by two-state models and *k*_obs_ from rapid mixing or temperature jump experiments varying linearly with ligand concentration (Shammas et al., 2013; Rogers et al., 2014b; Arai et al., 2015). Nevertheless, since both folding and binding take place, the underlying mechanism should be more complex than two-state. One of the processes involved may simply be too fast to be observed or be associated with too small a change in measurement parameters (fluorescence or NMR chemical shift, for example). Indeed, apparent two-state behavior is frequently observed in IDPs with simple bound topologies such as a single stretch of helix. In these cases, information on the binding mechanism and the role of pre-formed structure is often sought by modifying secondary structure content via mutation or by adding (secondary) structure stabilizing compounds such as trifluoroethanol (TFE; Gianni et al., 2012; Rogers et al., 2014b).

As discussed above, stabilizing the—in most studies, helical—bound-state secondary structure in IDPs has always been observed to stabilize the complexes with their binding partners, i.e., to increase affinity, while the effect of increased secondary structure on the on rate *k*_on_ of IDP binding is more ambiguous (Bienkiewicz et al., 2002; Gianni et al., 2012; Iešmantavičius et al., 2014; Rogers et al., 2014b; Arai et al., 2015). Nevertheless, in a majority of cases, *k*_on_ increases with increasing content of bound-state-like secondary structure within IDP binding sites, although to different extents. This behavior would be expected in a conformational selection scenario; increasing the population of the binding-competent conformer should lead to faster association. However, an increase in *k*_on_ strictly is only evidence for a reduced free energy of the rate-limiting transition state of the reaction with respect to the free state. This rate-limiting step may well correspond to folding occurring after initial binding; in that case, binding would be accelerated by the increased propensity of the binding site for the bound-state structure, rather than by increased availability of an obligatory pre-folded conformation (Iešmantavičius et al., 2014). Conversely, the observed complex stabilization by increased free-state secondary structure content has typically been traced to a reduction in the off rate (*k*_off_), i.e., a lower free energy of the final bound state with respect to the rate-limiting transition state (Gianni et al., 2012; Rogers et al., 2014a,b). Such an effect is normally interpreted as folding after binding, i.e., an induced fit mechanism. Again, however, this depends on where on the reaction coordinate the transition state occurs; it may correspond to a rate-limiting folding step that precedes binding. However, for binding reactions, it seems reasonable to assume that the transition state is already a complexed state; especially the formation of short segments of helical secondary structure in the free state of an IDP is unlikely to account for the rate-limiting step of a folding and binding interaction. Thus, if (de)stabilization of bound-state secondary structure affects *k*_off_, a contribution of an induced-fit mechanism to the binding can be assumed.

Similar to Φ value analysis that relates activation free energy to equilibrium free energy of binding (Fersht and Sato, 2004), information on the transition state of a folding and binding reaction can be obtained from how *K*_d_ varies with *k*_on_ or *k*_off_ for a series of modifying conditions (such as mutations or addition of TFE or denaturant; Prakash, 2011). If, for a given molecular system, a Brønsted plot of log(*k*_on_) or log(*k*_off_) against log(*K*_d_) is linear (i.e., gives a linear free-energy relationship, LFER), the position of the transition state on the reaction coordinate can be estimated from the slope of the plot. A large negative slope (approaching -1) in a plot of log(*k*_on_) against log(*K*_d_) indicates a “late” transition state with the property investigated by the modifications (such as secondary structure or tertiary contacts) largely formed, analogously to large values in Φ value analysis. Note that the results of such studies depend on the type of modification made to the system investigated. For example, for the interaction of the transcription factor c-Myb with the KIX domain of CBP, it has been postulated, based on kinetic binding studies in varying TFE concentrations, that c-Myb is largely unstructured in the transition state (Gianni et al., 2012). However, based on mutagenesis, it was concluded that the transition state exhibits a high degree (about 89%) of native order (Giri et al., 2013). It is difficult to envision how, in the transition state, the KIX binding region of c-Myb could be mostly unfolded and nevertheless undergo such a large percentage of interactions characteristic of the final bound complex, where it is present in a helical conformation (Zor et al., 2004). However, the mutations used were not chosen to perturb a specific quality such as secondary structure or hydrophobic interactions, and they were situated both within and outside of the binding interface, making their relative effects on folding and binding difficult to estimate. In the BH3 motif of disordered PUMA that binds as a helix to Mcl-1, secondary structure content was more specifically targeted by mutating residues outside of the binding interface to proline or glycine (Rogers et al., 2014a,b). Here, a modest effect on *k*_on_ (variation over one order of magnitude) was observed, but a much stronger one (up to 1000-fold) on *k*_off_, again interpreted as evidence for an early transition state and binding of PUMA in a mostly disordered conformation (Rogers et al., 2014a,b). These results indeed show that no particular part of the bound-state secondary structure of PUMA is strictly required for its binding. However, the free state of PUMA has been observed to exhibit only about 20% overall helicity; a transition state with little secondary structure is thus not yet evidence against conformational selection. Since the mutations employed did not vary helical content to a great extent, a small effect on *k*_on_ is perhaps not surprising. Also, some mutations introduced charge changes, and proline mutations are strongly disruptive to hydrogen bonding and may introduce additional complications via *cis-trans* isomerization.

In a study on the folding and binding of ACTR to the NCBD domain of CBP, helical secondary structure in free ACTR was targeted by carefully designed mutations to non-interface residues, explicitly excluding mutations to proline or involving changes in charge (Iešmantavičius et al., 2014). Resultant effects on helicity were monitored using both CD and NMR spectroscopy, and binding kinetics of the mutants to NCBD were measured by stopped-flow fluorimetry. Here, a clear correlation of free- state helical content, which varied in the range of 20–70% in the different mutants, with both *k*_on_ and *k*_off_, which both varied 2- to 2.5-fold, was found. The plot of log(*k*_on_) vs. log(*K*_d_) resulted in a slope of −0.47, while in an earlier study, mutations affecting the hydrophobic binding interface yielded an average Φ value of 0.14 (Dogan et al., 2013). This has been interpreted as a transition state in which crucial hydrophobic intermolecular interactions are only formed to 14%, but with the ACTR binding region already 47% helical (Iešmantavičius et al., 2014), indicating a mixed mechanism in which partly pre-formed conformers of ACTR bind and fold further to their final bound conformation within the complex. A reanalysis of the mutagenesis data performed in (Giri et al., 2013) in terms of the effect of the different mutations on helical secondary structure content also indicated that helicity is in fact correlated with *k*_on_ in the interaction of c-Myb with KIX (Arai et al., 2015).

### Mixed multistep mechanisms: A potential solution

Clearly, the different effects of mutations and cosolvents on folding and binding processes are often difficult to separate. However, an emerging consensus from this type of experiment seems to be that induced fit always has a role in the systems investigated, but that mixed mechanisms seem to be at play, as seen from the finding that pre-formed secondary structure affects on rates and is reflected in the degree of structuration of the transition state. It has already been noted that IDP binding mechanisms may be more complex than either conformational selection or induced fit, and that various combinations or intermediates of these two extremes need to be considered (Espinoza-Fonseca, 2009; Csermely et al., 2010). In particular, conformational selection and induced fit may coexist, with relative flux through the two pathways being determined by substrate concentration as well as the relative populations of the different forms of the protein undergoing a conformational change (Hammes et al., 2009). Reaction steps respectively exhibiting characteristics of conformational selection and induced fit may also occur sequentially in a multistep reaction (Espinoza-Fonseca, 2009; Csermely et al., 2010). Such complex behavior with parallel multistep pathways has been invoked in a detailed study for the folding and pyrophosphate binding of the *Bacillus subtilis* RNase P protein subunit (Daniels et al., 2014). Here, it was suggested that fast folding kinetics and low ligand concentrations favor conformational selection pathways, while slow folding and increasing ligand concentrations shift the balance toward induced fit pathways. With three conformational substates and two substrate binding sites, the system investigated in this study exhibits particular complexity; nevertheless, increasing evidence suggests that other IDPs may employ similar combinations of pathways and individual interaction steps in their interactions with binding partners.

As mentioned, an acceleration of binding by increased secondary structure content may reflect a rate-limiting folding step occurring after binding. However, there are indications for a role of partial conformational selection in such cases. As discussed above, very high, diffusion-limited association rates are recurrently found in interactions of IDPs with their partners, notably in the aforementioned PUMA–Mcl-1, cMyb–KIX, and ACTR–NCBD systems (Dogan et al., 2012; Rogers et al., 2013; Shammas et al., 2013). This is often cited as evidence for induced-fit type binding, since the requirement of a conformational selection mechanism for a pre-folded, possibly lowly populated, conformation should be incompatible with diffusion-limited association, where each encounter of the binding partners should lead to productive binding. However, it seems difficult to delineate the diffusion limit for disordered proteins, and even for induced-fit type binding, an energy barrier for folding after binding may remain that slows down the overall association (Rogers et al., 2013), likely in a similar way as conformational selection would. Most notably, however, the involvement of pre-formed structure in diffusion-limited interactions very likely depends on the degree of prestructuration of a particular molecule. For IDPs whose binding sites display a high content of secondary structure resembling the bound state, such as c-Myb(284–315) (70%; Arai et al., 2015) or the disordered C-terminal N_TAIL_ region of the nucleoprotein from Sendai virus (up to 75%; Jensen et al., 2008), it would likely be a pure induced-fit mechanism that would hamper fast association, given the prevalence of pre-formed conformers. It thus seems likely that, as described by Oas and coworkers for RNase P folding and binding (Daniels et al., 2014), highly populated, fast-folding pre-formed structure would preferentially participate in binding, at least if the partner protein is not present in excess.

After initial binding of pre-folded conformers, further folding may still occur within the complex. Such behavior is likely when secondary structure is significantly populated in the free state of an IDP, but is extended further in the final bound state, as seen for example in binding of the N-terminal region of the vesicular stomatitis virus (VSV) phosphoprotein P to the nucleoprotein N. Residues 25–31 of P form a transient helix populated up to 35% in the free state (Leyrat et al., 2012); in the N^0^P complex, P is helical in residues 17–31 (Leyrat et al., 2011). Similarly, in the transactivation domain of p53, residues 22–25 have been observed to populate helical secondary structure (Wells et al., 2008), while the crystal structure of the complex between this motif and the MDM2 oncoprotein features a helix in residues 19–25 (Kussie et al., 1996). Alternatively, the docking regions D1, D2, and D3 of the regulatory region of the mitogen-activated protein kinase (MAPK) kinase MKK7 all bind to the MAPK JNK1 in extended or polyproline conformation, despite sampling different regions of Ramachandran space in the free state (helical, polyproline, or random coil, respectively; Kragelj et al., 2015). For these interactions, an induced fit step appears obligatory since the bound-state conformation is not populated in the free ensemble, at least not to a measurable extent. Induced fit also certainly plays a role in the intriguing cases of individual disordered motifs binding to different partners in different conformations, as has been observed for residues 374–388 of the C-terminal regulatory region of p53 which bind to the proteins S100ββ, sirtuin, CBP, and cyclin A2 in helix, sheet, or different coil conformations, respectively (Oldfield et al., 2008). Induced fit has indeed been observed in an MD study of the interaction of S100ββ with p53 and another disordered partner, the TRTK-12 fragment from the protein CapZ. Here, MD simulations were complemented with Monte Carlo calculations for improved sampling of the binding process. Despite their differing sequences and bound-state structures, both peptides were observed to employ similar induced-fit binding mechanisms via non-native encounter complexes formed in the periphery of the S100ββ binding pocket. Nevertheless, helical structure similar to the bound state was observed in simulations of the free peptides (Staneva et al., 2012). Gō-type coarse-grained simulations of other IDP interactions such as those of PUMA with Mcl-1 or HIF1α, p53, and ACTR with their cognate domains of CBP (TAZ1, TAZ2, and NCBD, respectively) have also found important induced fit contributions to the binding mechanisms, involving a broad range of encounter complexes. Here, significant folding typically occurred only after formation of at least 20% of native intermolecular contacts (De Sancho and Best, 2012; Ganguly et al., 2013; Rogers et al., 2014a).

NMR studies of IDP folding and binding support the notion of complex multistep interactions with different contributions of conformational selection and induced fit. A seminal study of Wright and coworkers on the interaction of the pKID domain of CREB with the KIX domain of CBP suggested a model of exchange between four states, namely the free proteins, a transient, nonspecific encounter complex, a partially folded intermediate, and the final complex (Sugase et al., 2007a). The latter three states were inferred from fits to relaxation dispersion data measured on the complex, while the initial step of formation of an encounter complex was indicated by resonance signals of free pKID shifting in fast exchange during titration experiments. Encounter complex and intermediate were found to be only partially structured, clearly showing that this interaction proceeds largely via an induced-fit mechanism. A different result was obtained for the binding to KIX of c-Myb, whose primary binding site on KIX is the same as for pKID. Here, NMR relaxation dispersion data were consistent with an effective two-state mechanism (Arai et al., 2015), as was the case for stopped-flow and temperature-jump kinetic experiments (Gianni et al., 2012; Giri et al., 2013; Shammas et al., 2013). Based on the latter, an induced-fit mechanism was postulated (Gianni et al., 2012; Giri et al., 2013). However, recent coarse-grained as well as all-atom simulations of this interaction found no unique pathway for binding and indicated that c-Myb conformations with widely varying helical content can bind to KIX with similar probabilities (Ithuralde et al., 2016). In addition, in the transient kinetics experiments, a shorter c-Myb construct exhibiting relatively low helical content in the free state (around 30%) was used (Shammas et al., 2013). NMR data on the longer c-Myb (284–315) construct, on the other hand, rather indicated a fast free-state folding equilibrium. The large (70%) helical content in this construct, the effect on *k*_on_ of mutations affecting secondary structure, and the apparent high degree of structure in the transition state underline a role of conformational selection in this interaction (Giri et al., 2013; Arai et al., 2015). The differences apparent in the binding of pKID and c-Myb to KIX were related to their different roles in transcriptional activation, where largely unfolded KID requires induction by phosphorylation for high-affinity interaction with KIX, while the more helically preconfigured c-Myb acts as constitutive activator (Zor et al., 2002; Arai et al., 2015).

The previously mentioned case of the interaction of the ACTR activation domain with the NCBD domain of CBP has also been investigated by both non-equilibrium kinetics and NMR experiments, as well as Gō-type CG simulations. Here, an additional complication arises from the fact that both domains are disordered in their free states, however to different degrees. NCBD has been described as a molten globule (Kjaergaard et al., 2010), while ACTR is fully disordered with transient helical elements (Iešmantavičius et al., 2013). Both proteins thus undergo a coupled folding and binding reaction that has been described as mutual synergistic folding (Demarest et al., 2002). Free NCBD largely populates a conformation resembling its ACTR-bound state (Kjaergaard et al., 2010), while the H1 helix found in NCBD-bound ACTR is partly pre-formed in free ACTR to a population of 38% (Iešmantavičius et al., 2013). Transient kinetics experiments, as well as simulations, have suggested a multistep, largely induced-fit, binding mechanism (Dogan et al., 2012; Ganguly et al., 2013). However, as discussed above, the effects of mutations on binding kinetics have also indicated a partly helical transition state with few native tertiary interactions (Dogan et al., 2013) and a clear influence of helicity on association rate (Iešmantavičius et al., 2014). It thus seems likely that the pre-folded conformers present in the free-state ensembles of both ACTR and NCBD play a role in the initial binding step. For example, as has been suggested, the interaction may be initiated by a pre-formed first helix of ACTR making weak native-like contacts with the first helix of NCBD (Dogan et al., 2013). Such a mechanism is supported by the respective timescales of folding and binding observed in this system. NMR line shapes indicate that formation of the (fluid) tertiary structure of NCBD occurs on a timescale of 10^4^ s^−1^, with transient helix formation in ACTR likely even faster (Eaton et al., 1997; Jensen et al., 2008), while the rate of complex formation is on the order of 500 s^−1^ or slower (Iešmantavičius et al., 2014). Pre-formed binding competent conformers of both proteins would thus be readily available for complex formation.

**Figure 3:**
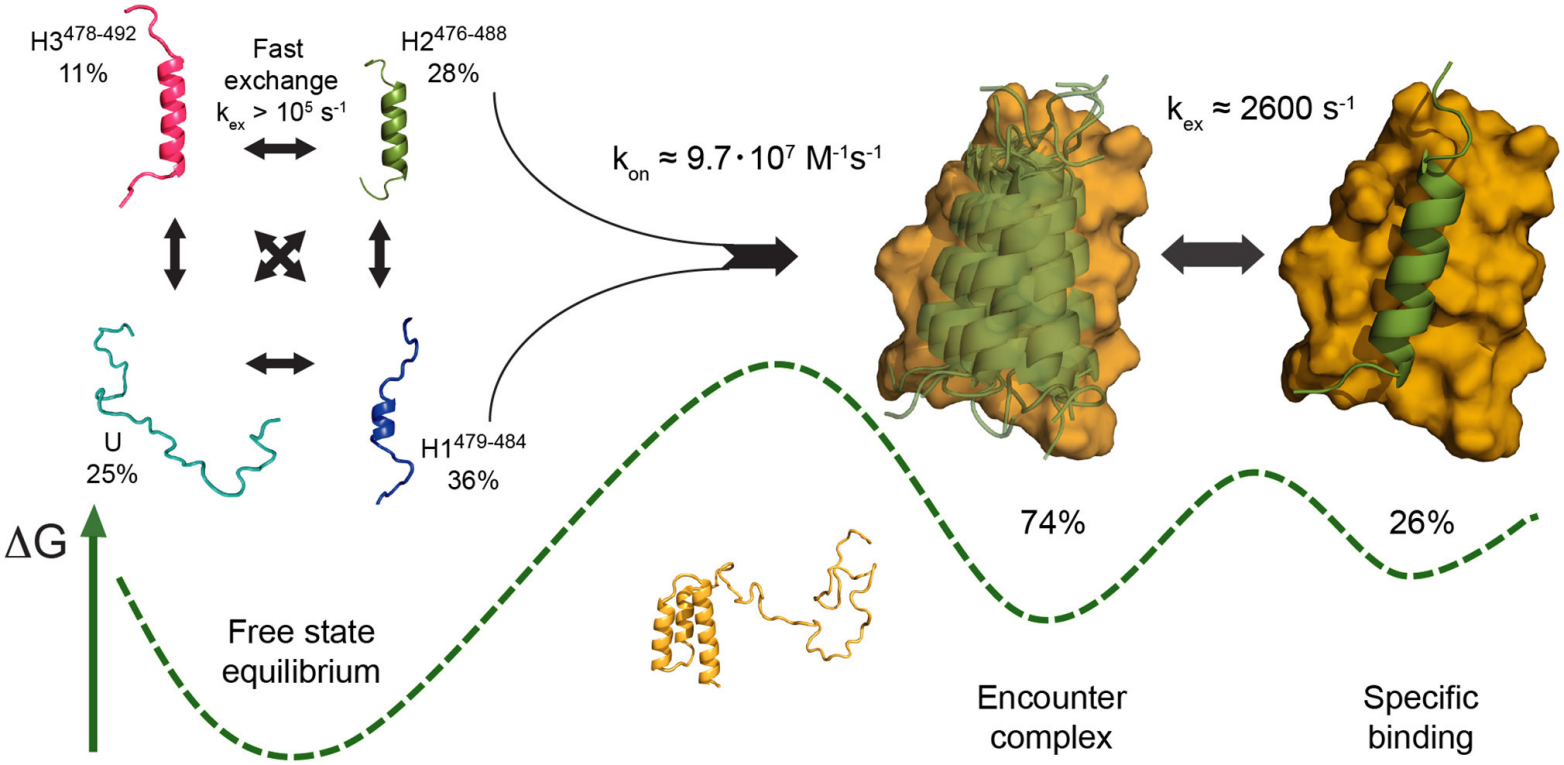
**Schematic of the mechanism of folding and binding of disordered Sendai virus N_**TAIL**_ to its partner PX, as proposed based on NMR relaxation dispersion data (Schneider et al., 2015), as an example of a multistep coupled folding and binding reaction with elements of both conformational selection and induced fit**. The PX binding site of free N_TAIL_ populates three defined helical conformers (H1–H3) in rapid exchange with a fully unfolded state (U) **(left)**. Interaction with PX stabilizes an N_TAIL_ conformer closely resembling H2 (green) in a nonspecific encounter complex on the surface of PX (yellow), likely involving conformational selection of H2 under the experimental conditions employed **(center)**. This N_TAIL_ conformer then locks into its final bound state in an interhelical groove of PX at a rate coincident with an intrinsic breathing motion of PX, a rearrangement step corresponding to induced fit **(right)**. A sketch of the free energy landscape is shown at the bottom, indicating the higher population of encounter complex compared to final complex deduced from experimental data. Adapted with permission from Schneider et al. (2015). Copyright 2015 American Chemical Society.

In another study relevant to the subject, we have investigated the interaction between disordered N_TAIL_ and the PX domain of the phosphoprotein from Sendai virus (SeV) using relaxation dispersion NMR experiments. The data were best explained by a three-state model in which one of the preexisting conformers of the highly helical free-state ensemble is initially stabilized in a nonspecific encounter complex with PX, as evidenced by the dominance of ^13^C backbone chemical shift modulations during the first step of the interaction. From a helical periodicity in ^15^N and ^1^H shifts that dominate the second step, we concluded that this N_TAIL_ helix then locks into its final bound position in a hydrophobic interhelical groove of PX, at a rate coincident with intrinsic motion of that groove (Schneider et al., 2015; Figure 3). The first step of this model thus comprises both folding and binding which could not be resolved kinetically. However, the bound conformation is—with one residue difference—nearly identical to one of the helical conformers populated to 28% in the free state. In addition, the molar fractions of PX used (<=15%) were below the population of that helical conformer, and the formation of helices in free N_TAIL_ is much faster—above 10^5^ s^−1^ from NMR data, in agreement with known helix folding rates (Eaton et al., 1997)—than the on rate, below 140 s^−1^ under the conditions measured. It can thus be assumed that, in these conditions, enough pre-folded N_TAIL_ conformers resembling the bound state will always be available for a conformational-selection type interaction in the first step of binding.

Interestingly, a temperature-jump kinetic study on the closely related N_TAIL_-XD system from measles virus (MeV) has also found complex kinetics with at least two steps, but has come to the opposite conclusion that the interaction is characterized by a global induced-fit mechanism (Dosnon et al., 2015). This study used the kinetic test in which the concentrations of both binding partners are varied in separate experiments (Gianni et al., 2014) and found a hyperbolic dependence of the observed rate constant *k*_obs_ in both cases, in agreement with an induced-fit interaction. The sequence homology between the corresponding proteins in the two viruses is rather low (about 20% identity), so the mechanisms involved in binding may well be different. However, it is unlikely that even the temperature jump technique, appropriate for fast reactions, is sensitive to the very fast rate of helix formation and interconversion in the free N_TAIL_ proteins of both viruses (Eaton et al., 1997; Jensen et al., 2008, 2011). The curvature observed in the variation of *k*_obs_ with the concentrations of both N_TAIL_ and XD from MeV confirms that a conformational change occurs within the complex between the two proteins. However, it is unknown what the observed change in tryptophan fluorescence corresponds to exactly at the molecular level. A “locking-in” of a pre-formed helical N_TAIL_ conformer on the surface of its binding partner, as inferred from NMR data on the SeV system, may also explain the unimolecular process detected in the temperature jump fluorimetry experiments on the MeV proteins. Thus, it appears likely that, in these two systems as well, conformational selection- and induced fit-type processes coexist in a sequential manner. The site-specific resolution offered by NMR spectroscopy clearly allows for additional detailed insight into the molecular processes underlying the measured kinetic parameters in these cases.

The MeV N_TAIL_-XD interaction has also been investigated by molecular simulations. Free N_TAIL_ was simulated using REMD, while simulation of the binding process either employed a hybrid potential, incorporating structural information on the bound state as attractive potential terms (Wang et al., 2013), or a combination of replica exchange and metadynamics (Han et al., 2016). Both studies reached similar conclusions about a sequential combination of conformational selection and induced fit in this system, in agreement with the experimental studies mentioned above. However, the composition of the simulated free-state ensemble in the study by Wang et al. differed somewhat from that of an ensemble based on experimental data (Jensen and Blackledge, 2014). In particular, experimental NMR RDCs were less well reproduced by the REMD ensemble, likely due to the prevalence of kinked helical conformers in the latter. The free-state ensemble described by Han et al. is similarly enriched in such structures, suggesting it is subject to the same issues regarding reproduction of experimental data. This observation underlines that current force fields still encounter problems in simulating the conformational sampling of IDPs in their isolated state, especially when transient structure is present. Consequently, caution should also be exercised in drawing conclusions on interaction mechanisms from molecular simulations.

### A synthetic view of IDP folding and binding

Overall, it appears that most coupled folding and binding reactions exhibited by IDPs are considerably more complex than could easily be described by either conformational selection or induced fit models. It has long been realized that these two mechanisms, while useful concepts and appropriate for the characterization of a range of known binding interactions, as well as individual reaction steps, are very likely too reductionist to describe the complexity of most biomolecular interactions entirely. The two limiting cases of flexible protein binding mechanisms seem to coexist not only as parallel pathways with flux-dependent relative importance (Hammes et al., 2009), but also sequentially in the multistep folding and binding reactions that are often found for IDPs. The picture that emerges from the studies discussed here is that conformational selection may play a role in the initial step of an IDP binding to its partner to a degree that corresponds to the amount of prestructuration present in its free-state ensemble. After the initial encounter, further induced-fit type rearrangements within the complex seem to occur nearly universally, from diffusion of a fully formed bound-state IDP conformation on the surface of its partner, as in the SeV N_TAIL_ –PX interaction (Schneider et al., 2015), to further folding of more complex structures, as apparent in, for example, the pKID–KIX or ACTR–NCBD interactions (Sugase et al., 2007a; Dogan et al., 2012).

Notably, the more complex a conformation adopted by an IDP in a complex, the more induced fit is likely to play a dominant role. While a single helix, given appropriate sequence composition such as N-capping residues (Richardson and Richardson, 1988; Doig et al., 1997; Jensen et al., 2008), can form easily and quickly, more complex topologies such as the three-helix fold of ACTR in complex with NCBD are unlikely to contribute significantly to initial binding in a folded form. Especially in the absence of a stable hydrophobic core, their spontaneous formation appears exceedingly unlikely; in addition, the steric hindrance of binding of such a pre-formed conformation to its partner would be considerable. In case of pure conformational selection, dramatically reduced association rates would be expected (Zhou et al., 2012). Rather, IDPs seem to achieve fast association by their ability to form complex bound topologies and extended binding interfaces rapidly by virtue of their flexibility, once the binding partner presents a template for their induced folding beyond a pre-formed folded “nucleus.” Coupled folding and binding of IDPs thus seems well described by the proposed “dock-and-coalesce” mechanism (Zhou et al., 2012) which, in terms classically used to describe protein folding, corresponds more to sequential structure formation as in diffusion-collision models (Karplus and Weaver, 1994), rather than nucleation-condensation mechanisms with concerted formation of secondary and tertiary structure (Itzhaki et al., 1995). Nevertheless, the variability observed in IDP binding mechanisms warrants caution in any such generalization.

In a broader sense, the observation that conformational selection and induced fit may be combined within a single pathway in a binding interaction suggests that the generic term “coupled folding and binding,” often employed to leave open the question about whether binding precedes folding or vice versa, may actually be a quite accurate description of more complex interactions of IDPs with their partners. It has been stated that concurrent folding and binding would be extremely improbable since, already individually, folding and binding are low frequency stochastic events whose simultaneous occurrence would thus be even more rare (Hammes et al., 2009). However, this assumes that folding and binding are instantaneous, purely random events occurring on a static energy landscape, which is likely not the case. In particular, it has to be kept in mind that the energy landscape of interacting molecules changes while they approach each other. This has been underlined by a recent all-atom MD study of the interaction of a folded protein, ubiquitin, with a short ubiquitin-interacting motif (UIM) peptide sequence (Long and Brüschweiler, 2011). Already at nanometer distance to the ligand, largely due to long-range electrostatic effects, it was found that the energy landscape of ubiquitin began to change, more and more favoring a preexisting energy well containing conformers similar to the bound state. It was pointed out that this interaction is well described by induced fit when considering the average structure of ubiquitin at any given protein-ligand distance; however, when considering the entire ensemble of ubiquitin conformations present at each point of the approach, conformational selection on a changing energy landscape is a more appropriate characterization. The overall mechanism was thus described as a “superposition” of conformational selection and induced fit (Long and Brüschweiler, 2011). Such a description may be well suited to explain the complex kinetics of folding and binding observed experimentally in IDPs, whose highly dynamic nature makes the need of ensemble descriptions even more evident (Jensen et al., 2014). Indeed, the term “conformational funneling” that we introduced in the context of the SeV N_TAIL_-PX interaction is conceptually very similar (Schneider et al., 2015; Gianni et al., 2016). This approach would also allow to transcend the likely too simplistic view of mutually exclusive, strictly sequential or strictly parallel conformational selection or induced fit pathways.

### Beyond structure: fuzzy complexes

While so far we have mostly discussed IDP binding interactions that involve individual binding sites and lead to well-defined complexes, the repertoire of binding mechanisms available to IDPs has been found to be much larger than that. So-called “fuzzy complexes” can retain considerable dynamics in the bound state (Tompa and Fuxreiter, 2008). In fact, the complex between N_TAIL_ and PX in Sendai virus discussed above already provides one example, with the nonspecific initial complex of N_TAIL_ diffusing on the surface of PX actually being more populated than the final bound state (Schneider et al., 2015). This dynamic behavior has also been used as an explanation for the persistent line broadening observed in NMR spectra of this complex, even in presence of excess ligand. The disordered cyclin-dependent kinase inhibitor Sic1 has been found to interact with a single binding site on its receptor Cdc4 via multiple phosphorylated suboptimal binding sites that engage the partner in rapid exchange and only become transiently ordered upon interaction (Mittag et al., 2008). The requirement for each site to be phosphorylated for interaction, as well as the rapid equilibrium of several Sic1 sites exchanging on a single receptor binding site, leads to global high-affinity binding and a finely tunable, sensitive response of this interaction to Sic1 phosphorylation. An even more extreme example of dynamic binding is found in the interaction of phenylalanine-glycine- (FG-) rich nucleoporins (FG-Nups) with nuclear transport receptors (NTRs) during their transit through the nuclear pore complex. A recent detailed investigation of the interaction of the NTR importin β with a PxFG-rich domain of the FG-Nup Nup153 has demonstrated extremely rapid, concurrent binding of minimalistic Nup153 motifs (in principle, individual phenylalanine side chains) to importin β, while the overall disordered nature of Nup153 remained unperturbed, to the extent that backbone ^13^C chemical shifts remain oblivious to the interactions mediated by their aromatic sidechain moieties (Milles et al., 2015). The unique properties of this multivalent interaction have been proposed to be at the core of rapid nuclear transport. Dynamic complexes of IDPs and multisite interactions thus seem to provide important advantages for rapid, yet sensitive and selective molecular recognition, with the avoidance of a large entropy loss upon binding likely being one of them. The hypothesis that an IDP interaction could be dynamic to such an extent that NMR spectra remain completely unaffected, as put forward for the dimerization of the T-cell receptor zeta subunit (Sigalov et al., 2007), has however not been confirmed by further experiments (Nourse and Mittag, 2014), suggesting that even “fuzzy complexes” are characterized by a transient local gain in structure within binding sites.

## Concluding remarks

IDPs often seem to escape concepts that attempt to unify and generalize their behavior. While some phenomena, such as fast association rates or moderate affinities, are found recurrently in IDP interactions, they are not generally associated with disorder, and IDPs appear to employ various combinations of conformational selection and induced fit mechanisms of binding, making use of both pre-formed structured elements and structural adaptation after binding. However, it should not be expected that IDPs are a homogeneous class of proteins; their mechanistic repertoire is large, as is the range of functions they are involved in. In that sense, IDPs should probably be regarded as less fundamentally different from folded proteins than they appear at first glance; after all, they are governed by the same fundamental laws of kinetics and thermodynamics. In recent years, techniques such as transient kinetics, NMR spectroscopy, and molecular simulations have considerably increased our knowledge about the mechanisms employed by IDPs to fulfill their functions. In particular, it has become increasingly evident that combining the results gained using these different techniques allows for additional mechanistic insight not easily obtained from the individual approaches alone. Further research in this exciting field should allow us to gain a more representative picture on what may or may not distinguish disorder from order in protein function.

## Author contributions

All authors participated in writing of the manuscript and approved of its final version.

### Conflict of interest statement

The authors declare that the research was conducted in the absence of any commercial or financial relationships that could be construed as a potential conflict of interest.
